# FAMILIAR CONTEXTS, FAMILIAR EMOTIONS? A NEW PERSPECTIVE ON CONTEXT-SPECIFIC EMOTION PROCESSES IN OLDER ADULTHOOD

**DOI:** 10.1093/geroni/igac059.1441

**Published:** 2022-12-20

**Authors:** Tabea Springstein, Tammy English

**Affiliations:** Washington University in St. Louis, St. Louis, Missouri, United States; Washington University in St. Louis, St. Louis, Missouri, United States

## Abstract

As people age, their emotional well-being tends to be maintained or improves. Theories of adult development suggest that features of the context (e.g., more familiar environments) and successful management of emotions contribute to this effect. This talk focuses on how familiarity may promote emotional differentiation (e.g., knowing whether one feels angry or sad) in daily life, an important predecessor to emotion regulation success. A sample (N=290) of community participants between the ages of 25 and 85 years old completed an experience sampling study (6x/10days). When people were more familiar with their current situation, they differentiated more between emotions. The relationship between familiarity and differentiation was stronger for older adults than younger adults. These results support the perspective that emotional well-being benefits from wisdom accrued over the lifespan, such that older adults are at more of an advantage when their daily contexts afford for them to draw on their prior experiences.

